# *In Vitro* and *In Vivo* Antileishmanial Effects of *Pistacia khinjuk against Leishmania tropica* and *Leishmania major*

**DOI:** 10.1155/2015/149707

**Published:** 2015-02-28

**Authors:** Behrouz Ezatpour, Ebrahim Saedi Dezaki, Hossein Mahmoudvand, Mojgan Azadpour, Fatemeh Ezzatkhah

**Affiliations:** ^1^Razi Herbal Medicines Research Center, Lorestan University of Medical Sciences, Khorramabad 6814994688, Iran; ^2^Leishmaniasis Research Center, Kerman University of Medical Sciences, Kerman 7616914111, Iran

## Abstract

The present study aims to evaluate the* in vitro* and* in vivo* antileishmanial activities of* Pistacia khinjuk *Stocks (Anacardiaceae) alcoholic extract and to compare its efficacy with a reference drug, meglumine antimoniate (MA, Glucantime), against* Leishmania tropica *and* Leishmania major*. This extract (0–100 *µ*g/mL) was evaluated* in vitro* against promastigote and intracellular amastigote forms of* L. tropica* (MRHO/IR/75/ER) and then tested on cutaneous leishmaniasis (CL) in male BALB/c mice with* L. major* to reproduce the antileishmanial activity topically.* In vitro*,* P. khinjuk *extract significantly (*P* < 0.05) inhibited the growth rate of promastigote (IC_50_ 58.6 ± 3.2 *µ*g/mL) and intramacrophage amastigotes (37.3 ± 2.5 *µ*g/mL) of* L. tropica* as a dose-dependent response. In the* in vivo* assay, after 30 days of treatment, 75% recovery was observed in the infected mice treated with 30% extract. After treatment of the subgroups with the concentration of 20 and 30% of* P. khinjuk* extract, mean diameter of lesions was significantly (*P* < 0.05) reduced. To conclude, the present investigation demonstrated that* P. vera *extract had* in vitro* and* in vivo* effectiveness against* L. major*. Obtained findings also provide the scientific evidences that natural plants could be used in the traditional medicine for the prevention and treatment of CL.

## 1. Introduction

Cutaneous leishmaniasis (CL) is caused by the transmission of* Leishmania* spp. through the bite of female sandfly. This disease is characterized by chronic skin lesions and leaves permanent scars as deformation of the infected area [1]. CL is a public health problem with the annual incidence rate of 1.5 million people throughout the world. According to World Health Organization's (WHO) report, 12 million people are infected by parasites and 350 million people are living in regions with the high risk of infection [2]. In Iran, both epidemiological forms of this skin disease are present: anthroponotic CL (ACL) and zoonotic CL (ZCL) caused by* Leishmania tropica* and* Leishmania major*, respectively [3]. At present, the current medications used to treat leishmaniasis such as meglumine antimoniate and sodium stibogluconate are a challenge due to having problems of emerging resistance, high toxicity, or high costs [4]. Since there is no effective vaccine, maintenance and improvement of existing treatment regimens, combined with new drug discovery initiatives, were found to be the only ways to guarantee continued control of CL [5].

Natural products, such as plants extract, either as pure compounds or as standardized extracts, provide unlimited opportunities for new drug discoveries because of the unmatched availability of chemical diversity [6, 7]. The genus* Pistacia* belongs to the family Anacardiaceae. Among 15 known species of pistachios, only 3 species grow in Iran, which include* P. vera*,* P. khinjuk*, and* P. atlantica* [8].* P. khinjuk* Stocks commonly grows in the Mediterranean and Middle Eastern countries for the last 3000 years. In Iran, this plant is called “khenjuk or kelkhong” and grows in the central, western, and eastern areas [9]. Different parts of the plant including resin, leaf, bark, fruit, and aerial parts have been used widely as traditional medicine for the treatment and prevention of various disease conditions such as stomach discomfort, nausea, vomiting, and motion sickness [10]. In addition, reviews have reported* P. khinjuk* to have anti-inflammatory, antioxidant, antitumor, antiasthmatic, and antimicrobial properties [8]. The present study aims to evaluate the* in vitro* and* in vivo* antileishmanial activity of* P. khinjuk* extract and to compare its efficacy with a reference drug, meglumine antimoniate (MA, Glucantime) against* L. tropica* and* L. major*.

## 2. Materials and Methods

### 2.1. Chemicals

MA as a control drug was purchased from Aventis, France. Penicillin and streptomycin were obtained from Alborz Pharmacy, Karaj, Iran, and stored at room temperature (25°C) until testing. MTT powder [3-(4.5-dimethylthiazol-2-yl)-2.5-diphenyl tetrazolium bromide], fetal calf serum (FCS), fetal bovine serum (FBS), RPMI-1640 medium with L-glutamine, and Griess reagent (A and B) were prepared from Sigma-Aldrich (St. Louis, MO, USA). All other chemicals and solvents had the highest commercially available purity.

### 2.2. Collection of Plant Materials

**Figure 1:**
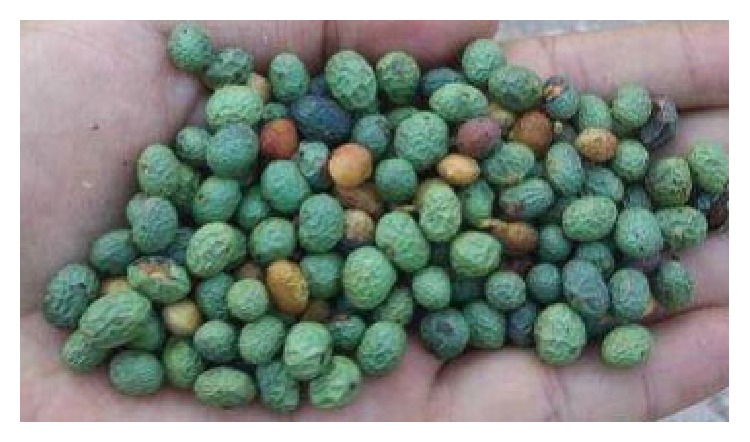
*Pistacia khinjuk* fruit which is called “kelkhong” in Iran.

Fruits of* P. khinjuk* were collected from rural regions of Kerman province, south east of Iran, from May to September 2013 (Figure 1). They were identified by a botanist of Botany Department of Shahid Bahonar University of Kerman, Kerman, Iran. A voucher specimen of the plant materials was deposited at the herbarium of Department of Pharmacognosy, School of Pharmacy, Kerman University of Medical Sciences, Iran (KF 1135).

### 2.3. Preparation of Extract

Air-dried plant materials (100 g) were separately extracted by percolation method with 70% aqueous ethanol successively for 72 h at room temperature. The extracts were passed through filter paper (Whatman number 3, Sigma, Germany) to remove plant debris. The extracts were finally concentrated in vacuum at 50°C using a rotary evaporator (Heidolph, Germany) and stored at −20°C until testing [11].

### 2.4. Phytochemical Analysis

The preliminary phytochemical analysis of the* P. khinjuk* ethanolic extract was carried out to determine the presence of tannins, saponins, alkaloids, flavonoids, and glycosides as described elsewhere using the following reagents and chemicals [12]: alkaloids with Mayer and Dragendorff's reagents, flavonoids with the use of Mg and HCl, tannin with 1% gelatin and 10% NaCl solutions, glycosides with FeCl_2_ and H_2_SO_4_, and saponin with ability to produce suds.

### 2.5. Parasite and Cell Culture

Standard strains of* L. tropica* (MHOM/IR/2002/Mash2) and* L. major* (MRHO/IR/75/ER) were kindly prepared from Center for Research and Training in Skin Diseases and Leprosy, Tehran, Iran. The parasites were cultured at RPMI 1640 supplemented with penicillin (200 IU/mL), streptomycin (100 *μ*g/mL), and 15% heat-inactivated FCS. Murine macrophage cells (J774-A1) were obtained from Pasteur Institute of Iran, Tehran, Iran. The cells were cultured and maintained at Dulbecco's modified eagle's medium (DMEM) supplemented with 10% FBS at 37°C in 5% CO_2_.

### 2.6. Cytotoxic Effects

In order to determine the cytotoxic effects of* P. khinjuk*, J774-A1 cells (5 × 10^5^) were cultivated with various concentrations of alcoholic extract (0 to 5000 *μ*g/mL) in 96-well tissue culture plates at 37°C in 5% CO_2_ for 48 h. Cell viability was determined by colorimetric MTT assay and the results were displayed as the percentage of dead cells compared to macrophages treated with MA and nontreated macrophages (100% of viability) [13].

### 2.7. *In Vitro* Antileishmanial Activity

#### 2.7.1. Antipromastigote Assay

To evaluate the antipromastigoteeffects of* P. khinjuk* ethanolic extract against promastigote forms of* L. tropica*, colorimetric cell viability MTT assay was used as described elsewhere [14]. At first, 100 *μ*L of the promastigotes (10^6^ cells/mL) harvested from logarithmic growth phase was added to a 96-well tissue culture plate. Then, 100 *μ*L of various concentrations of alcoholic extract and MA (0–100 *μ*g/mL) was added to each well and incubated at 25 ± 1°C for 48 h. After the incubation, 10 *μ*L of MTT solution (5 mg/mL) was added to each well and incubated at 25°C for 4 h. Promastigotes were cultured at complete medium with no drug as positive control and complete medium with no promastigotes and drugs as blank. The absorbance was measured for each well at 560 nm using an ELISA reader (BioTek-ELX800, USA).

#### 2.7.2. Antiamastigote Assay

In this study, antiamastigote activity of* P. khinjuk* extract was performed using the methods described by Mahmoudvand et al. [15]. Briefly, before adding the macrophages to the plates, 1 cm^2^ cover slips were placed in the wells of 6-chamber slides (Lab-Tek, Nalge Nunc International, NY, USA). In the next step, 200 *μ*L of macrophage (J774) cells (10^5^/mL) was incubated at 37°C in 5% CO_2_ for 2 h. Then, 200 *μ*L (10^6^/mL) promastigotes in stationary phase was added to the murine macrophages so that proportion of* Leishmania*/macrophage was 10 : 1 and incubated again in a similar condition for 24 h. Free parasites were removed by washing with RPMI 1640 medium and the infected macrophages were treated with 50 *μ*L of various concentrations of 0–100 *μ*g/mL alcoholic extract at 37°C in 5% CO_2_ for 72 h. Finally, the dried slides were fixed with methanol, stained by Giemsa, and studied under a light microscope. Also, the macrophages containing amastigotes without extract and those with no parasite and extract were considered positive and negative controls, respectively. Activity of anti-intramacrophage amastigotes of the extracts was evaluated by counting the number of amastigotes in each macrophage by examining 100 macrophages (% amastigotes viability) in comparison with those obtained by positive control.

#### 2.7.3. Inhibition of Infection in Macrophage Cells

The inhibitory effect of* P. khinjuk* extract against the promastigotes invasion of macrophages, as one of the most important pathogenic and biological criteria of* Leishmania* parasites, was investigated. In this assay, promastigotes of* L. tropica* were preincubated in* P. khinjuk* extract (5 *μ*g/mL) for 2 h at room temperature. Then promastigotes were washed with RPMI-1640 medium and incubated with macrophage cells for 24 h. The macrophages were stained by Giemsa and studied by a light microscope, to evaluate the frequency of infection by counting 100 macrophages [3].

#### 2.7.4. Nitric Oxide Production Determination

In this study, nitric oxide (NO) release in supernatants of macrophage culture was measured by the Griess reaction for nitrites compared to the untreated macrophages. One hundred *μ*L of supernatants was collected 48 h after introducing the various concentrations of* P. khinjuk* extract (3.125, 6.25, and 12.5) into the culture medium. The assay was done in triplicate wells in a 96-well tissue culture plate. To this, 60 *μ*L of Griess reagent A and then 60 *μ*L of Griess reagent B were added. The plates were read at 540 nm in an ELISA plate reader (BioTek-ELX800, USA).

### 2.8. *In Vivo* Antileishmanial Activity

#### 2.8.1. Ethical Statement

The experimental procedures carried out in this survey were in compliance with Guidelines of Kerman University of Medical Science (Kerman, Iran) for the care and use of laboratory animals in line with Animal Ethics Committee (permit number 91/21).

#### 2.8.2. Animals

Thirty-two male BALB/c mice (6–8 weeks old) were obtained from Animal Breeding Stock Facility, Razi Institute of Iran, Karaj, Iran. The animals were housed in a colony room with a 12 : 12 h light/dark cycle at 21 ± 2°C and handled according to standard protocols for the use of laboratory animals [16].

#### 2.8.3. Infecting of BALB/c Mice by Injecting of* L. major*

**Figure 2:**
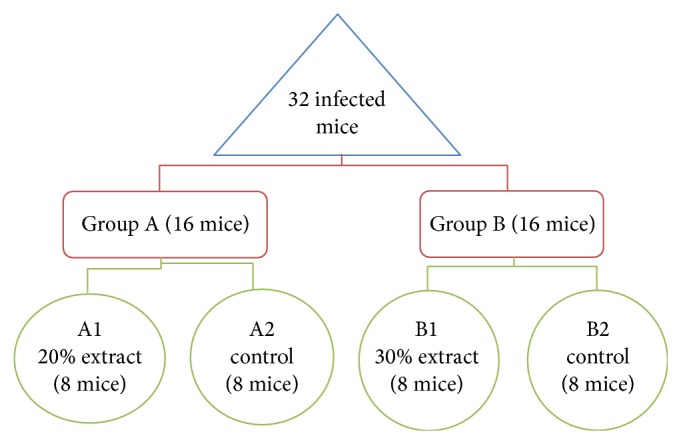
Flow chart of* in vivo* antileishmanial activity of* P. khinjuk* alcoholic extract after 30-day application as intralesionally compared to meglumine antimoniate (MA) as control drug.

To infect the male BALB/c mice, 0.1 mL of the promastigotes of* L. major* (2 × 10^6^ cells/mL) harvested from the stationary phase was subcutaneously injected into the base of the tail [17]. Thirty-two mice were divided into two groups; then, every group was divided into two subgroups; so, every group contained eight mice (Figure 2).

#### 2.8.4. Treatment of Infected Mice

After 5 weeks when leishmanial lesions appeared, the treatment of infected mice was started. At first, the diameter of lesions was measured before the treatment. Then, lotion (5 g/kg ethanolic extract at the concentrations of 20 and 30%) and MA were applied for each tested subgroup daily for 30 days topically. The control subgroups received distilled water and ethanol topically.

#### 2.8.5. Measurement of Lesion Size

Before and after the treatment, the diameter of lesions was measured using vernier caliper in two diameters (*D* and *d*) at right angles to each other, and the size (mm) was determined according to the formula *S* = (*D* + *d*)/2 [18].

#### 2.8.6. Microscopical Examinations to Determine Parasite Load

Microscopical examinations were performed by laboratory demonstration of the parasite load in the lesions by making stained smears at the end of the experimental period. Lesions were cleaned with ethanol and punctured at the margins with a sterile lancet and exudation material was smeared. The smears were dried in air, fixed by methanol, and stained with Giemsa to determine the load of parasites by light microscopy as follows:

 negative: 0 parasite/10 fields,
 1+: 1 parasite/10 fields,
 2+: 1–10 parasites/10 fields,
 3+: 10–100 parasites/10 fields,
 4+: 1010–1000 parasites/10 fields,
 5+ and more: more than 1000 parasites/10 fields.

### 2.9. Statistical Analysis

In this survey, all the tests were carried out in triplicate. To determine CC_50_ (cytotoxic concentration for 50% of cells) and IC_50_ (50% inhibitory concentrations) lineal regression was used. Selectivity index (SI), calculated based on the equation of CC_50_ for murine macrophage cells/IC_50_ for amastigote forms of* L. tropica*, was used to compare the toxicity and activity of ethanolic extract of* P. khinjuk*, as described by Weniger et al. [19]. Data analysis was carried out using SPSS statistical package, version 17.0 (SPSS Inc., Chicago, IL, USA). Differences between the test and control groups were analyzed by *t*-test. In addition, *P* < 0.05 was considered statistically significant.

## 3. Results

### 3.1. Phytochemical Analysis

In this study, the findings of primary phytochemical screening of the ethanolic extract of* P. khinjuk* demonstrated the presence of high amount of tannins, phenols, and glycosides and lacking of the alkaloids in this plant.

### 3.2. *In Vitro* Antileishmanial Effects

#### 3.2.1. Antipromastigote Activity

The results revealed that* P. khinjuk* alcoholic extract had remarkable antileishmanial activity against the promastigote forms of* L. tropica* based on a dose-dependent response (*P* < 0.05). IC_50_ value for the* P. khinjuk* extract and MA against promastigotes of* L. tropica* was 58.6 ± 3.15 *μ*g/mL and 88.3 ± 4.05 *μ*g/mL, respectively.

#### 3.2.2. Antiamastigote Assay

The findings demonstrated that, similar to promastigote stage,* P. khinjuk* extract significantly (*P* < 0.05) inhibited the growth rate of intramacrophage amastigotes as a dose-dependent response. The obtained IC_50_ values were 37.3 ± 2.51 *μ*g/mL and 44.6 ± 2.51 *μ*g/mL for the* P. khinjuk* extract and MA, respectively.

#### 3.2.3. Inhibition of Infection

The results revealed that promastigote forms of* L. tropica* with no drugs were able to infect 84.3 ± 6.5 percent of macrophage cells. While promastigotes treated with the* P. khinjuk* had potency to infect only 38.6 ± 3.05 percent of the macrophages cells, thus, these findings revealed that infectivity of promastigotes of* L. tropica* significantly reduced (*P* < 0.05) with* P. khinjuk* extract preincubation.

#### 3.2.4. NO Production Determination

The findings of this assay demonstrated that the* P. khinjuk* at concentration of 3.125 *μ*g/mL triggered production of 14.3 ± 1.5 *μ*M of nitric oxide compared to the untreated macrophages with value of 11.3 ± 0.5 *μ*M (*P* > 0.05), whereas this extract at higher concentrations (≥6.25 *μ*g/mL) decreased production of NO to ≤7 *μ*M, compared to the untreated macrophages (*P* > 0.05).

#### 3.2.5. Cytotoxic Effects

In the assessment of* in vitro* cytotoxic activity of alcoholic extract of* P. khinjuk*, it could be observed that* P. khinjuk* extract had no significant cytotoxicity against J774 cells. CC_50_ values for* P. khinjuk* extract and MA were 511.6 ± 7.15 *μ*g/mL and 1225.6 ± 11.6, respectively. SI values for the alcoholic extract* P. khinjuk* and MA are 13.7 and 27.5, respectively.

### 3.3. *In Vivo* Antileishmanial Effects

**Figure 3:**
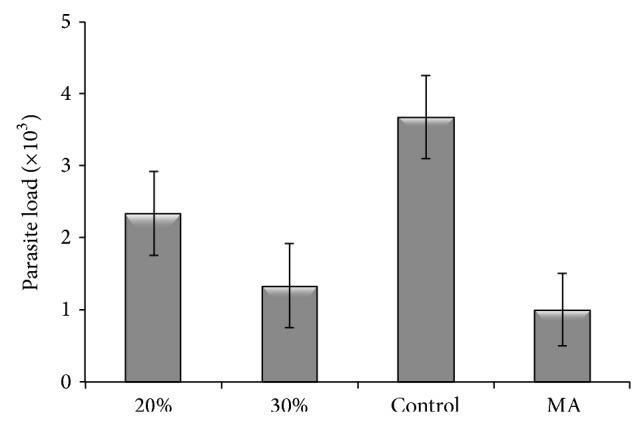
Comparison of parasite load in tested groups after treatment with various concentrations of* P. khinjuk* alcoholic extract.

*In vivo* antileishmanial evaluation of the* P. khinjuk* alcoholic extract demonstrated that, in the infected mice treated with the extract concentration of 30%, the number of parasites significantly (*P* < 0.05) reduced with respect to MA, whereas 20% of the extract decreased the number of parasites intermediately. Control subgroups (distilled water and ethanol) had no decrease in the number of parasites (Figure 3). After 30 days of treatment, 75 and 87.5% recovery were observed in the infected mice treated with 30% extract and MA, respectively, while* P. khinjuk* extract at the concentration of 20% recovered 50% of the infected mice. After treatment of the subgroups with the concentration of 20%* P. khinjuk* extract, mean diameter of lesions was reduced to 0.19 cm. In contrast, in the subgroups treated with 30%* P. khinjuk* extract, mean diameter of lesions was decreased to 0.44 cm. In the positive subgroups, mean diameter of the lesions was reduced to 0.77 cm (Table 1).

## 4. Discussion

According to WHO, more than 80% of the world's population rely on traditional medicine for their primary healthcare needs. In the past decades, the advent of synthetic antimicrobial drugs has caused reluctance in plants as a rich resource for antimicrobial agents [20] However, in recent years, the emergence of some limitations in the use of these drugs has caused changes in the situation and interest in the field of ethnobotanical research [21]. In the present investigation, the* in vitro* and* in vivo* antileishmanial activities of alcoholic extract of* P. khinjuk* were assessed against* L. tropica* and* L. major*. The obtained results of optical density (OD) and consequently IC_50_ values demonstrated that* P. khinjuk* alcoholic extract significantly inhibited the growth rate of promastigote forms of* L. tropica*. In addition, this extract significantly decreased mean infection rate and subsequently the viability of amastigote forms in the macrophages compared with the control group. The findings exhibited that amastigote forms were more susceptible to* P. khinjuk* extract than promastigote forms. This difference in the susceptibility of promastigote and amastigote forms could be related to their structural, biochemical, and morphological features as previously demonstrated by other researchers [22, 23]. In the* in vivo* assay, it was found that* P. khinjuk* extract at the concentration of 30% had potent suppression effects on CL in male BALB/c mice infected with* L. major* with 87.5% recovery, whereas* P. khinjuk* extract at the concentration of 20% displayed the suppression effects as intermediate. In line with our results, it has been proven that gum obtained* P. atlantica* controlled cutaneous leishmaniasis in mice infected with* L. major* [24]. These results were in agreement with those of the previous studies, indicating that the commonly used herbs had antimicrobial properties that, in some cases, can be used in traditional medicine [25]. Previously, in several studies, antibacterial, antiviral, and antifungal effects of different parts of* Pistacia* species, particularly* P. khinjuk*, have been demonstrated [8]. However, there are few studies on the antiparasitic effects of* Pistacia* species including the study conducted by Orhan et al., which proved that* P. vera* branch extract at the concentration of 4.8 *μ*g/mL significantly (77.3%) inhibited the growth of* L. donovani*, whereas dry leaf extract (PV-DL) was active against* Plasmodium falciparum* (60.6% inhibition) [26]. This research also revealed that the IC_50_ values of these extracts were determined as 2.3 *μ*g/mL for the amastigotes of* L. donovani* grown at axenic culture and 3.65 *μ*g/mL for* P. falciparum*.

The preliminary phytochemical analysis of* P. khinjuk* extract demonstrated the presence of terpenoids, phenols, flavonoids, fatty acids, and sterols and lack of alkaloids in this plant. At present, individual activities of these compounds have been proven [20]. Moreover, different studies have shown potent antileishmanial activities of these compounds such as terpenic derivatives, carvacrol,* p*-cymene, thymol, carvone, limonene, and terpinene [27–30]. Therefore, the presence of these phytoconstituents in* P. khinjuk* extract could be responsible for their antileishmanial effect though their exact mode of action is not clear. In the case of cytotoxic effects, the present findings showed that* P. khinjuk* alcoholic extract had no significant cytotoxic effects against J774 cells. Furthermore, SI value ≥ 10 of* P. khinjuk* alcoholic extract demonstrated its safety against the macrophage cells and specificity to the parasite [19]. Thus, it can be suggested that* P. khinjuk* alcoholic extract is safe for mammalian cells.

In conclusion, the findings of the present study demonstrated that* P. khinjuk* alcoholic extract had potent antileishmanial activity and could control cutaneous leishmaniasis in mice infected with* L. major*. This result also provided the scientific evidence that natural plants could be used in the traditional medicine for the prevention and treatment of cutaneous leishmaniasis.

## Figures and Tables

**Table 1:** Effect of various concentrations of *P. khinjuk* alcoholic extract before and after treatment on the size of lesions (cm) in Balb/c mice infected by *L. major*.

Concentrations	Size of lesions (cm) before treatment	Size of lesions (cm) after treatment	*P* value^b^
20%	1.31 ± 0.29	1.12 ± 0.20	<0.05
30%	1.28 ± 0.29	0.84 ± 0.12	<0.05
MA^a^	1.37 ± 0.22	0.42 ± 0.12	<0.05
Control	1.23 ± 0.25	2.09 ± 0.26	—

^a^Meglumine antimoniate.

^b^Difference is significant when compared to control.
